# A Universal Scaling Relation for Defining Power Spectral Bands in Mammalian Heart Rate Variability Analysis

**DOI:** 10.3389/fphys.2018.01001

**Published:** 2018-08-02

**Authors:** Joachim A. Behar, Aviv A. Rosenberg, Ori Shemla, Kevin R. Murphy, Gideon Koren, George E. Billman, Yael Yaniv

**Affiliations:** ^1^Faculty of Biomedical Engineering, Technion-IIT, Haifa, Israel; ^2^Cardiovascular Research Center, Division of Cardiology, Rhode Island Hospital, Warren Alpert Medical School of Brown University, Providence, RI, United States; ^3^Department of Physiology and Cell Biology, The Ohio State University, Columbus, OH, United States

**Keywords:** heart rate variability, animal models, mammals, power spectral analysis, power allometric law

## Abstract

**Background:** Power spectral density (PSD) analysis of the heartbeat intervals in the three main frequency bands [very low frequency (VLF), low frequency (LF), and high frequency (HF)] provides a quantitative non-invasive tool for assessing the function of the cardiovascular control system. In humans, these frequency bands were standardized following years of empirical evidence. However, no quantitative approach has justified the frequency cutoffs of these bands and how they might be adapted to other mammals. Defining mammal-specific frequency bands is necessary if the PSD analysis of the HR is to be used as a proxy for measuring the autonomic nervous system activity in animal models.

**Methods:** We first describe the distribution of prominent frequency peaks found in the normalized PSD of mammalian data using a Gaussian mixture model while assuming three components corresponding to the traditional VLF, LF and HF bands. We trained the algorithm on a database of human electrocardiogram recordings (*n* = 18) and validated it on databases of dogs (*n* = 17) and mice (*n* = 8). Finally, we tested it to predict the bands for rabbits (*n* = 4) for the first time.

**Results:** Double-logarithmic analysis demonstrates a scaling law between the GMM-identified cutoff frequencies and the typical heart rate (HR_m_): f_VLF-LF_ = 0.0037⋅${\mathrm{HR}}_{m}^{0.58}$, f_LF-HF_ = 0.0017⋅${\mathrm{HR}}_{m}^{1.01}$ and f_HFup_ = 0.0128⋅${\mathrm{HR}}_{m}^{0.86}$. We found that the band cutoff frequencies and Gaussian mean scale with a power law of 1/4 or 1/8 of the typical body mass (BM_m_), thus revealing allometric power laws.

**Conclusion:** Our automated data-driven approach allowed us to define the frequency bands in PSD analysis of beat-to-beat time series from different mammals. The scaling law between the band frequency cutoffs and the HR_m_ can be used to approximate the PSD bands in other mammals.

## Introduction

The HR is controlled by dynamic and chaotic processes, and it oscillates at different periods over continuously shifting time scales (Goldberger et al., 1990). Therefore, even under resting conditions, mammalian electrocardiographic (ECG) recordings exhibit complex beat-to-beat variations (Billman et al., 2015b). Analysis of the beat-to-beat variation of the HR, a field of research known as HRV analysis, can reveal information about the underlying physiological processes that control its dynamics. For example, a loss of complexity in HRV has been documented in several cardiovascular diseases and has been correlated with an increase in morbidity and mortality (Hillebrand et al., 2013), while abnormal beating patterns can be characteristic of arrhythmias such as atrial fibrillation (Behar J.A. et al., 2013).

Power spectral density (PSD) analysis of the HR fluctuation provides a quantitative non-invasive means for HRV and for assessing the function of the cardiovascular control system (Akselrod et al., 1981). In healthy humans, the frequency of the PSD performed on a 5-min long recording is traditionally divided into three main bands (Malik et al., 1996): the high frequency (HF) band, 0.15–0.4 Hz, where the dominant HF peak can typically be found around 0.25–0.3 Hz; the low frequency (LF) band, 0.04–0.15 Hz, where the dominant peak can typically be found around 0.1 Hz; and the very low frequency (VLF) band, 0.0033–0.04 Hz. The HF band corresponds to rhythms with periods between 2.5 and 7 s (McCraty et al., 2001) and is known to reflect parasympathetic (vagal) activity and respiratory sinus arrhythmia (Akselrod et al., 1981). The LF band corresponds to rhythm modulations with periods between 7 and 25 s and is believed to mainly reflect baroreflex activity while at rest (Malliani, 1995). The LF was also often assumed to have a dominant sympathetic component, but this assumption has been strongly challenged (Billman, 2013b). The VLF corresponds to rhythms with periods between 25 and 300 s. VLF power has been strongly associated with all-cause mortality in cohorts with cardiac failure or multiple organ dysfunction syndrome (Tsuji et al., 1996; Hadase et al., 2004; Schmidt et al., 2005). The energy contained in this band has been suggested to be intrinsically generated by the heart (Kember et al., 2000, 2001). These frequency bands for humans were standardized by the Task Force of the European Society of Cardiology (Malik et al., 1996) based on the review of years of empirical evidence from various studies (Akselrod et al., 1981; Malik et al., 1996), which applied frequency analysis of the HRV and observed regions of interest within the PSD.

Mammals are commonly used for cardiovascular research. Dogs, rabbits and mice have been of particular interest: dogs are physiologically close to humans and thus a reliable experimental model to study cardiac diseases (Hasenfuss, 1998). The rabbit is the smallest mammal with Ca^2+^ dynamics similar to humans (Bers, 2002; Terentyev et al., 2014; Morrissey et al., 2017). With the recent advances in genome manipulation technologies, there has been increased interest in using animals with mutations designed to overexpress or knock out genes implicated in human cardiovascular diseases (Thireau et al., 2008). Mice have been of particular interest in that regard (Tzimas et al., 2017; Hook et al., 2018). Because of the physiological differences across mammals, a quantitative approach is necessary for adapting the frequency bands to different mammalian electrophysiological data. Defining mammal-specific frequency bands is necessary if the PSD analysis of the HR time series is to be used as a proxy for measuring the autonomic nervous system (ANS) activity in animal models.

One parameter that can be used to adapt frequency band cutoffs to different mammals is the HR, and one possible mathematical formulation of such scaling law is the power law. The existence of such a law has not been studied in the context of adapting PSD parameters in HRV analysis, specifically between PSD band cutoff frequencies from different mammals and their respective HR_m_. Identifying such a power law would enable approximating the PSD frequency bands to mammals other than the ones analyzed in this work.

It has been shown that an allometric law exists between the body mass (BM_m_) and various biological processes, such as metabolic rate, life span, HR_m_, and the ECG PR interval (West et al., 1997, 1999; Noujaim et al., 2004). This scaling is described by the equation Y = Y _0_ ⋅ BM^b^, where *Y* is the biological process and *b* is the scaling exponent. Whether an allometric scaling law exists between the PSD band cutoff frequencies and the BM_m_ has not been reported. A secondary aim of this research is to explore whether such an allometric law exists.

This work aims to: (1) create an automated data-driven approach to identify appropriate frequency bands for power spectral analysis of the beat-to-beat time series obtained from different mammalian ECG data; (2) use the estimated bands to research a universal power law between PSD band cutoff frequencies and the typical heart rate (HR_m_) of different mammals. This power law could be used to approximate the frequency bands for any other mammal; (3) explore whether a power allometric law exists between the BM_m_ and the characteristic PSD bands.

## Materials and Methods

### Mammal Databases and Ethical Approval

We used ECG recordings from healthy humans (*n* = 18) (Goldberger et al., 2000), heartworm-free mixed-breed dogs (*n* = 17) (Billman et al., 2015a), C57BL/6 male mice (*n* = 8) (Yaniv et al., 2016), and New Zealand white rabbits (*n* = 4) (Brunner et al., 2008; Odening et al., 2012).

The public access MIT-BIH Normal Sinus Rhythm (MIT-NSR) database was used for human data (Goldberger et al., 2000). This database was chosen because it contains long ECG recordings of humans having no known cardiovascular condition. Thus, it can be used to define the baseline bands for human HRV analysis. The dog experiments (Billman et al., 2015a) were approved by the Ohio State University Institutional Animal Care and Use Committee and conformed to the Guide for the Care and Use of Laboratory Animals (revised 1996) published by the National Academies Press (Washington, DC, United States). The mouse data (Yaniv et al., 2016) were obtained in accordance with the Guide for the Care and Use of Laboratory Animals published by the National Institutes of Health (NIH Publication no. 85-23, revised 1996). Experimental protocols were approved by the Animal Care and Use Committee of the National Institutes of Health (protocol #441LCS2013). The rabbit data (Brunner et al., 2008; Odening et al., 2012) were recorded in accordance with the local guidelines of the institutions and only after approval by the Institutional Animal Care and Use Committee of Pennsylvania State University College of Medicine and the Milton S. Hershey Medical Center, Hershey, PA, United States, and the Institutional Animal Care and Use Committee of Rhode Island Hospital, Providence, RI, United States, in accordance with the NIH Guide for the Care and Use of Laboratory Animals (NIH Publication no. 85-23, revised 1996). All animal data were obtained from healthy, free-moving animals.

Human data were recorded at 128 Hz, dog data at 500 Hz, and rabbit and mouse data at 1 kHz. All the mammals were conscious, had no known cardiac condition, and no drugs were administered previous to the ECG recordings. For the animal databases, we performed peak detection (Behar et al., 2014) to identify the R-peak locations and we computed the RR interval (defined as the time variation between consecutive R-peaks) time series. We excluded from the dataset large segments of noise. We defined noise segments as the inability of a human annotator to clearly identify R-peaks on the raw ECG trace (Behar J. et al., 2013). Only transient sections of noise were left in the database. In addition, the R-peaks were manually corrected when necessary in order to ensure the reliability of the RR time series. **Table 1** summarizes the mammalian databases used in this study. Supplementary Tables S1–S3 provide more details on the individual recordings for each mammal.

**Table 1:** Mammalian electrophysiological database.

	Human	Dog	Rabbit	Mouse
Number of records	18	17	20	8
Number of mammals	18	17	4	8
Total recordings length (hr:min:sec)	437:29:36	01:33:55	03:31:13	03:28:07
Median RR interval (msec)	766	484	239	109
95% RR interval (msec)	359 – 1117	360 – 896	210 – 301	87 – 156

*Data from humans (Goldberger et al., 2000), dogs (Billman, 2013a), rabbits (Brunner et al., 2008; Odening et al., 2012), and mice (Yaniv et al., 2016) were used.*

### Processing the RR Intervals

Before performing PSD analysis, the RR intervals must be preprocessed to ensure that only beats from normal sinus node depolarizations are used. In order to filter out non-sinus beats, we used a moving average filter. We chose the window size to be identical to the values used by the PhysioNet HRV toolkit (Mietus and Goldberger, 2017): a moving average filter with a window size of 21 samples and RR intervals exceeding 20% (for humans, rabbits and mice) or 30% (for dogs) of the window’s average were removed. The 20–30% thresholds were empirically chosen. In humans, a threshold of 20% is usually used (Mietus and Goldberger, 2017) and it was suitable for the rabbit and mouse data. However, with the dog dataset the 20% threshold was too stringent and removed a high number of normal sinus beats. Thus we decided to select a threshold of 30%, which was more suitable for this dataset. The NN time series is defined as the preprocessed RR time series using the moving average filter.

### Power Spectral Density Analysis

The upper bound of the HF band (f_HFup_) defines the minimal resampling frequency of the NN time series (i.e., f_s_ ≥ 2 ⋅ f_HFup_, following Shannon’s criterion). In the literature, f_HFup_ was set to 0.4 Hz in humans (Malik et al., 1996), but there is no clear rationale for this cutoff. To look for this cutoff we computed the PSD up to the maximal frequency meaningful to resolve (f_max_), which was set as the frequency corresponding to the ‘characteristic shortest RR interval’ found in normal sinus rhythm ECG recordings. We defined the ‘characteristic shortest RR interval’ as the lower bound of the interval containing 95% of the RR intervals from a large population. We used the histograms of the RR intervals computed on our databases to obtain these values. Given f_max_, we estimated the PSD on the interval [0-f_max_] Hz using the Welch periodogram (Welch, 1967). Because Welch PSD estimation requires uniformly sample data, the RR interval time series was resampled at 2.2 times f_max_. The resampling frequency was chosen such that it satisfies Shannon’s criterion

To perform PSD analysis, a window (i.e., sub-segment) of the NN time series must be selected. This window must be long enough to resolve the VLF band and short enough to assume data stationarity (Malik et al., 1996). The traditional window for humans is 5 min long (Malik et al., 1996). Because no alternative window length has been standardized for dogs and rabbits, we used the same window size as for humans (i.e., 5 min). We used a 3 min window for mice, as suggested in Thireau et al. (2008). We denote as ‘VLF’ the frequency band starting from 0.0033 Hz for humans, dogs and rabbits [corresponding to the lowest frequency we can resolve using a 5 min window size 1/(60^∗^5)], and 0.0056 Hz for mice [corresponding to the lowest frequency we can resolve using a 3 min window size 1/(60^∗^3)], up to the upper bound of the VLF band. In summary, non-overlapping 5 min windows were used for human, dog and rabbit data and non-overlapping 3 min windows were used for the mouse data. PSD was computed for each non-overlapping window. In the instances where the recording was less than the window size length, the PSD was computed on the total recording length available. This happened in particular for the dog data, where a number of recordings were 4–5 min long.

**FIGURE 1:**
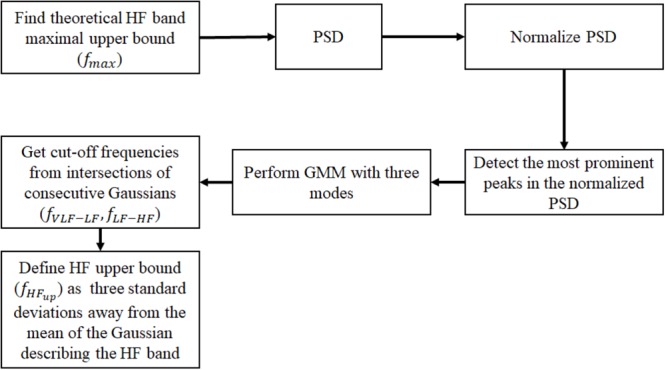
Block diagram of the algorithm for identifying the frequency bands from a database of mammalian NN intervals. PSD, power spectral density; GMM, Gaussian mixture model; VLF, very low frequency; LF, low frequency; HF, high frequency.

For each non-overlapping window, we normalized the PSD by the total power in order to allow cross-comparison of mammal types. For each normalized PSD, we detected prominent frequency peaks. Given the location of the detected peaks for each window size, we created a histogram of the prominent peak locations for each mammal type (see example in Supplementary Figure S1). We assumed that the histograms were generated from a mixture of Gaussian distributions; thus, we used a GMM (Reynolds, 2015) to estimate the Gaussian parameters that best describe the underlying distribution. Because of the three traditional power spectral bands (VLF, LF and HF), we used three Gaussians for the GMM (thus assuming three modes). We estimated the GMM and defined the intersection between consecutive Gaussians as the band cutoff frequencies. To set the minimal peak height threshold (defined as the minimal amplitude a peak must have in the normalized PSD to be considered as ‘prominent’), we used the human data. Given the selected minimal peak height threshold for humans, we applied the same algorithm to the dog, rabbit and mouse data. We defined the upper bound of the HF (f_HFup_) band as three standard deviations away from the mean of the Gaussian describing the HF band. Note that f_max_ defined the upper frequency bound up to which we compute the PSD, whereas f_HFup_ corresponds to the final upper cutoff frequency of the HF band as estimated from the GMM approach. A block diagram summarizing the main steps to identify the frequency bands is shown in **Figure 1**. We trained the algorithm on a database of human ECG recordings (*n* = 18) and validated it on databases of dogs (*n* = 17) and mice (*n* = 8). Finally, we tested it to predict the bands for rabbits (*n* = 4) for the first time.

### Power-Law

Given the bands identified by the GMM for humans, dogs, rabbits, and mice, we looked for a power-law relationship between the band cutoff frequencies or mean of each Gaussian and HR_m_. HR_m_ was defined as the median HR evaluated on our database for a given mammal. To search for power laws between band cutoff frequencies and HR_m_, we used a double-logarithmic analysis of the band cutoffs for each mammal type against HR_m_. To search for a power law between the dominant peak in each of the GMM- identified band and HR_m_, we used a double-logarithmic analysis of the dominant peak location for each 5- or 3-min segment against the mean HR of the segment. We used linear regression to explore whether a linear relationship existed between the variables in the double-logarithmic plot.

### Power Allometric Law

To explore this, we used a double-logarithmic analysis of the band cutoffs for each mammal type against the typical BM_m_ of the mammals included in our database. The typical BM_m_ values for the different mammals were taken from Noujaim et al. (2004). We used linear regression to explore whether a linear relationship existed between the variables in the double-logarithmic plot.

## Results

### Power Spectral Band Definition

**FIGURE 2:**
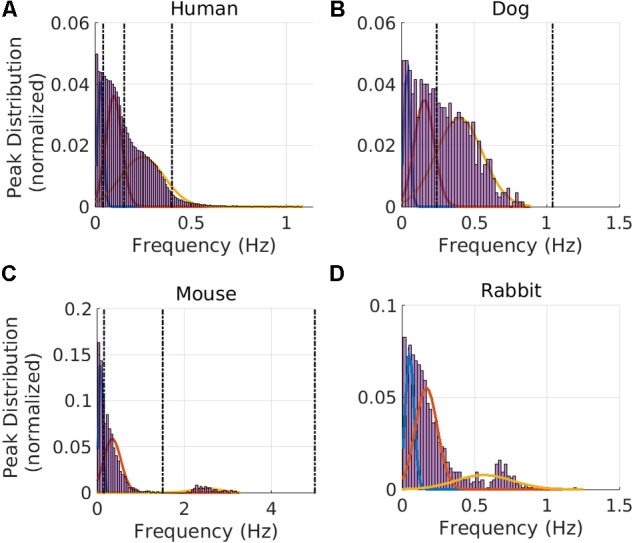
Gaussian Mixture Model and frequency band clustering. Cutoff frequency identification for the VLF, LF, and HF regimes for **(A)** humans, **(B)** dogs, **(C)** mice, and **(D)** rabbits. The vertical dotted line corresponds to values used in the literature: human (Malik et al., 1996), dog (Billman, 2013a; Billman et al., 2015b), and mouse (Thireau et al., 2008). For dogs, no VLF-LF cutoff could be found in the literature, so only the LF-HF and f_HFup_ vertical lines from the literature are shown. The intersection between two consecutive Gaussians defines the band cutoffs.

We used the human ECG data to train our GMM. The median RR interval for humans was 766 ms and the lower bound of the RR distribution (thus describing the ‘fastest’ heart rate) was 359 ms (see **Table 1**). Therefore, f_max_ was set to 2.8 Hz. Based on GMM fitting, the VLF band was identified to be between 0.0033 and 0.046 Hz, the LF band between 0.046 and 0.158 Hz, and the HF band between 0.158 and 0.588 Hz. **Figure 2A** shows the GMM estimated by overlaying the estimated Gaussians onto the histogram of the prominent peak locations.

We next validated our results on data from dogs and mice. Using the GMM model trained on the human data, the bands were estimated for the dog data; the VLF band was found to be between 0.0033 and 0.067 Hz, the LF band between 0.067 and 0.235 Hz, and the HF band between 0.235 and 0.877 Hz. **Figure 2B** shows the GMM estimated by overlaying the estimated Gaussians onto the histogram of the prominent peak locations. Using the GMM model trained on the human data, the bands were estimated for the mice data; the VLF band was identified to be between 0.0056 and 0.152 Hz, the LF band between 0.152 and 1.240 Hz, and the HF band between 1.240 and 3.471 Hz. **Figure 2C** shows the GMM estimated by overlaying the estimated Gaussians onto the histogram of the prominent peak locations.

Using the GMM approach trained on the human data, the bands were estimated for the rabbit data; the VLF band was identified to be between 0.0033 and 0.088 Hz, the LF band between 0.088 and 0.341 Hz, and the HF band between 0.341 and 1.155 Hz. **Figure 2D** shows the GMM estimated by overlaying the estimated Gaussians onto the histogram of the prominent peak locations.

In addition to the frequency band cutoffs, we also report the Gaussian means and standard deviations for each band of each mammal (**Table 2**). Finally, we computed the power ratio: the relative power of each band over the total power for each mammal (**Table 3**). Our data show that the HF power is relatively higher in larger mammals (humans and dogs) than in smaller mammals (rabbits and mice).

**Table 2:** Gaussian mean (μ) and standard deviations (σ).

	Human (μ ± σ)	Dog (μ ± σ)	Rabbit (μ ± σ)	Mouse (μ ± σ)
G_V LF_	0.026 ± 0.015	0.035 ± 0.020	0.049 ± 0.030	0.074 ± 0.046
G_LF_	0.095 ± 0.042	0.154 ± 0.068	0.165 ± 0.077	0.339 ± 0.198
G_HF_	0.246 ± 0.114	0.397 ± 0.160	0.564 ± 0.197	2.505 ± 0.322

*Numbers are rounded to the third decimal place. *G*_*VLF*_, the Gaussian describing the VLF band, *G*_*LF*_ the Gaussian describing the LF band, and *G*_*HF*_ the Gaussian describing the HF band.*

**Table 3:** Power ratio (of relative power over total power, %) for the different bands.

	Human	Dog	Rabbit	Mouse
VLF (%)	55.5	40.0	71.5	63.8
LF (%)	28.0	20.6	19.4	25.8
HF (%)	19.0	40.7	10.4	11.0

### Power Law and Allometric Power Law

**FIGURE 3:**
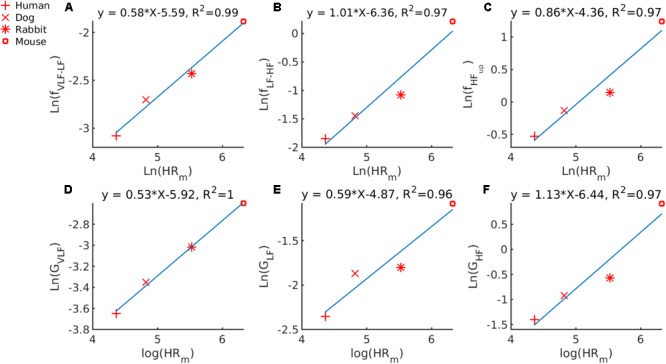
Double-logarithmic plot of PSD band cutoff frequency and Gaussian means versus median HR_m_ for **(A)** f_VLF-LF_ the cutoff frequency between the VLF and LF bands versus the HR_m_; **(B)** f_LF-HF_, the cutoff frequency between the LF and HF bands versus the HR_m_; **(C)** f_HFup_, the upper bound of the HF band versus the HR_m_; **(D)** G_V LF_, the mean of the Gaussian describing the VLF band; **(E)** G_LF_, the mean of the Gaussian describing the LF band, and **(F)** G_HF_, the mean of the Gaussian describing the HF band.

First, we used our GMM approach for defining the frequency bands to determine whether a universal scaling relation exists between HR_m_ and the PSD band cutoff frequencies between VLF and LF (f_VLF-LF_) or LF and HF f_LF-HF_or the upper bound frequency of the HF band (f_HFup_). By examining the median HR_m_ for humans and mice in our database, we obtained a median HR_m_ that ranges from 78 to 550 bpm. Similarly, f_VLF-LF_ranges from 0.046 to 0.152 Hz, f_LF-HF_ ranges from 0.158 to 1.240 Hz and f_HFup_ from 0.588 to 3.471 Hz. In **Figure 3A**, we plotted ln(f_V LF-HF_) against ln(HR_m_). The cutoff frequency scales with the HR_m_ following the power law: f_VLF-LF_ = 0.0037 ⋅ ${\mathrm{HR}}_{m}^{0.58}$. In **Figure 3B**, we plotted ln(f_LF-HF_) versus ln(HR_m_). Thus, f_LF-HF_ scales with HRm as f_LF-HF_ = 0.0017 ⋅ ${\mathrm{HR}}_{m}^{1.01}$. In **Figure 3C**, we plotted ln(f_HF_up__) versus ln(HR_m_). Thus, f_HF_up__ scales with HR_m_ following the power law: f_HF_up__ = 0.0128 ⋅ ${\mathrm{HR}}_{m}^{0.86}$.

Second, we used our GMM approach to determine whether a universal scaling relation exists between HR_m_ and the mean of the 0027⋅${\mathrm{HR}}_{m}^{0.53}$, G_LF_ = 0.0077 ⋅ ${\mathrm{HR}}_{m}^{0.59}$ and G_HF_ = 0.0016 ⋅ ${\mathrm{HR}}_{m}^{113}$.

Third, we tried to determine whether a universal scaling relation exists between the HR and the dominant PSD peak in each of the frequency bands. In Supplementary Figure S2 we plotted ln(peak_band_) for each frequency band (VLF, LF and HF) versus ln(HR). Our data indicate that only the peak_HF_ is correlated with the HR. The dominant peak in the HF band scales with HR as peak_HF_ = 0.0019 ⋅ HR^1.09^.

**FIGURE 4:**
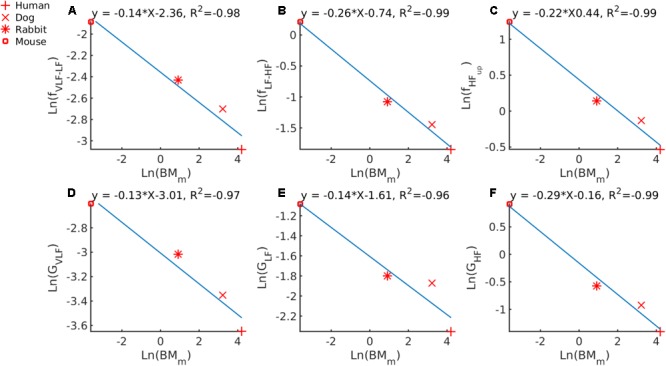
Double-logarithmic plot of PSD band cutoff frequencies and Gaussian means versus BM_m_ for **(A)** VLF to LF cutoff versus BM_m_, **(B)** LF to HF cutoff versus BM_m_, **(C)** upper bound of HF versus BM_m_. Center of the Gaussian describing **(D)** the VLF band versus BM_m_, **(E)** the LF band versus BM_m_ and **(F)** the HF band versus BM_m_.

Fourth, we tried to determine whether a universal law for allometric scaling in biology also exists between BM_m_ and the band cutoff frequencies identified by the GMM. We first checked that we could retrieve with our data the known -1/4 allometric law between HR_m_ and BM_m_ as reported in West et al. (1997). Supplementary Figure S3 illustrates the result of the line fit for this analysis. The scaling power found on our data was -0.24, which is very close to -1/4, as expected. From the linear fit (**Figures 4A–C**) the following power-law relationships were found: f_VLF-LF_ = 0.0944 ⋅ ${\mathrm{BM}}_{m}^{-0.14}$, f_LF-HF_ = 0.4771 ⋅ ${\mathrm{BM}}_{m}^{-0.26}$ and f_HFup_ = 0.644 ⋅ ${\mathrm{BM}}_{m}^{-0.22}$.

Finally, we tried to determine whether a universal law for allometric scaling in biology also exists between BM_m_ and the mean of the Gaussians describing each band (**Table 2**). In **Figure 4D**, we plotted G_V LF_ against ln(BM_m_) and an allometric law of G_V LF_ = 0.0493 ⋅ ${\mathrm{BM}}_{m}^{-0.13}$ was found. In **Figure 4E** we plotted ln(G_LF_) against ln(BM_m_) and an allometric law of ln(G_LF_) = 0.1999 ⋅ ${\mathrm{BM}}_{m}^{-0.14}$ was found. In **Figure 4F** we plotted ln(G_HF_) against ln(BM_m_) and an allometric law of ln(G_HF_) = 0.8521 ⋅ ${\mathrm{BM}}_{m}^{-0.29}$ was found.

## Discussion

Our first major contribution is the introduction of a new data-driven approach to identify the frequency bands in PSD analysis of heartbeat interval time series from different mammals. To the best of our knowledge, this is the first attempt to provide an automated and data-driven approach for defining the frequency bands in PSD analysis of mammalian HRV. For the human data, our algorithm identified the bands as: VLF [0.0033 – 0.046] Hz, LF [0.046 – 0.158] Hz and HF [0.158 – 0.588] Hz. These results are comparable to the recommended bands (Malik et al., 1996) (VLF [0.0033 – 0.04] Hz, LF [0.04 – 0.15] Hz and HF [0.15 – 0.4] Hz), thus validating our approach.

Our second major contribution is the application of the method to dog, rabbit and mouse ECG data. Using ECG data from healthy and awake animals (**Figure 2**), we showed that the frequency cutoffs between the VLF-LF and LF-HF bands could be defined. We validated our model by comparing its results to bands experimentally identified in the literature for dogs and mice: the cutoff between LF and HF in dogs data is similar to the one used in the work of Billman (2013a). Similar cutoffs to ours are also documented for mice: the cutoff between VLF and LF and between LF and HF is similar to other works (Ishii et al., 1996; Just et al., 2000; Joaquim, 2004; Tankersley et al., 2004; Campen, 2005; Fazan, 2005; Adachi et al., 2006; Farah et al., 2006; Baudrie et al., 2007; Thireau et al., 2008). Finally, we tested our model on the rabbit dataset. One study (Goldstein et al., 1995) has looked into defining frequency bands in rabbits. However, this study was performed on anesthetized rabbits, which may require different band definitions than the ones for conscious rabbits, particularly because of the changes in HR that can be caused by the different agents used and the depth of anesthesia (Picker et al., 2001; Mazzeo et al., 2011). Thus, we defined for the first time the HRV frequency bands for this mammal. The prominent peak found in the HF band is characteristic of the respiratory rate (respiratory sinus arrhythmia) (Akselrod et al., 1981). Therefore, we expect the mean of the Gaussian describing the HF band to fall within the respiratory range of the corresponding mammal. The values identified by the GMM (0.397, 0.564, and 2.505 Hz for dogs, rabbits and mice, respectively) fall within the respiratory rate range for these three mammals: 20–40 breaths per minute (brpm) for dogs [0.33–0.67] Hz, 30-60 brpm for rabbits [0.5–1] Hz, and 60–220 brpm for mice [1–3.67] Hz (University Wisconsin-Madison, 2018). Thus the GMM approach successfully retrieved the vagal activity enabled by respiratory effort and which is known to manifest in the HF band.

Our third major contribution is the definition of the upper bound of the HF bands. The cutoff values found in the literature are based on empirical observations. We defined the upper bound of the HF band as being three standard deviations away from the mean of the Gaussian representing the HF band. The upper boundary of the HF band for humans was 0.588 Hz. This is higher than the recommended one (0.4 Hz) (Malik et al., 1996). This higher-than-recommended boundary is likely due to our use of the entire 24 Holter ECG in the MIT NSR database. Indeed, the 24 Holter ECG might include periods of exercise in which the breathing rate might be higher than 24 brpm, i.e., 0.4 Hz. It thus seems reasonable to allow a higher HF upper bound than the standard recommended cutoff for humans. Using the same method, the cutoff frequencies defined for the other mammals were 0.877, 1.155, and 3.471 Hz for dogs, rabbits, and mice, respectively.

Interestingly, our results show that the mouse may serve as a better mammalian model than do the dog or rabbit for studying the effects of drugs, mutations, or cardiac diseases on vagal activity as reflected in the HF band. Indeed, the Gaussian fittings for mice show minimal overlap between the LF and HF bands (**Figure 2**) compared to other mammals. This means that the vagal activity in mice can be studied without interference from the physiological processes echoed in the low frequency band. However, this is moderated by the larger HF power ratio for humans and dogs (19 and 40.7% respectively) versus rabbits and mice (10.4 and 11% respectively, **Table 3**). This means that rabbits and mice display relatively less vagal activity than humans and dogs. The effect of respiration is dominant in the HF band. However, during periods of slow respiration, the resulting vagal activity will modulate the HR at frequencies which will cross over into the LF band (Ahmed et al., 1982; Tiller et al., 1996). Thus, for a breathing rate lower than 9.6 brpm, the characteristic vagal frequency peak will fall in the LF band in humans. We interpret this observation as being due to the higher breathing rates of smaller mammals, which leads to a better separation between the respiratory sinus arrhythmia modulation of the heart rate [reflected mainly in the HF band (Supplementary Figure S4)] and other autonomic nervous system effects, partly reflected in the LF bands.

Our fourth major and most important contribution is our discovery of a power-law relationship between band cutoff frequencies, dominant PSD peak, and HR_m_. Thus, a relationship exists between our GMM-identified cutoff frequencies and HR_m_. This scaling law can be used to approximate the PSD bands for other mammals not included in our study. In order to define the bands for non-human mammals, other works (Yaniv et al., 2014) scale the frequency bands used in humans linearly with the ratio of human HR_m_ and HR_m_ of another mammal. **Figure 3** shows that this approach is incorrect because the relationship between the frequency cutoffs and the HR_m_ is not linear with respect to the ratio of the mean heart rates, as the equations for the log-log subplots highlight. Interestingly, a scaling relation could only be found between the dominant HF peak and HR (Supplementary Figure S2). Because the HF peak represents vagal activity and because the scale power was 1, our results suggest that vagal activity in different mammals is linearly associated with HR. The prominent peak found in the HF band shifts with changes in the respiratory rate (Akselrod et al., 1981), and thus a scaling law was shown between the respiratory rate and the typical HR across mammals. We tested whether the power law that we found can be used to approximate the HRV band cutoff frequencies from other species than the ones included in this work. Aubert et al. (1999) have investigated frequency bands in experimental rat models using direct manipulation of autonomic parameters through pharmacological intervention. They identified the LF and HF bands as: LF [0.19 – 0.74] Hz and HF [0.78 – 2.5] Hz. The mean HR of the rat population used by Aubert et al. (1999) was 345 bpm. Using this typical HR value and the power law we established between HR_m_ and the PSD cut-off frequencies, we predicted the bands as: VLF [0.0033 – 0.110] Hz, LF [0.110 – 0.622] Hz and HF [0.622 – 1.949] Hz, which is close to the experimental findings of Aubert et al. (1999).

Our fifth major contribution is our discovery that the band cutoff frequencies in mammals, f_LF-HF_ and f_HFup_, follow an allometric law that scales as the ∼-1/4 power of the body mass and that G_V LF_ and G_LF_ scale as the ∼-1/8 power of the body mass. Interestingly, the 1/4 scaling power has been shown to be an essential component in biological scaling (West et al., 1997). In the model of West et al. (1999), processes that rely on hierarchical networks for resource distribution are identified to scale with ${\mathrm{BM}}_{m}^{n/4}$ (with *n* 𝜖 [scale=.60]./fig/img001.eps). In practice, many variables in mammalian physiology have been shown to scale with BM_m_ following such a quarter-power law. These include metabolic rate, circulation time, HR, aortic diameter, and respiratory rate (West et al., 1997; Noujaim et al., 2004). Because the frequency bands correspond to the regulation of specific physiological processes (such as baroreflex or vagal activity) on the HR dynamic, it was interesting to find that the band cutoff frequencies (f_LF-HF_, f_HFup_) and the BM_m_ also followed this universal quarter allometric scaling to ensure optimal autonomic activity in regulating the HR dynamics.

### Limitations

Although the total length of the recordings was acceptable for the different mammals (**Table 1**), the sample size (i.e., the number of animals) was limited for mice and rabbits. Moreover, the data were obtained here for specific species of mammals and breeds; however, the scaling laws found can be used to approximate the bands given the typical HR_m_ of another species. For a more precise estimation of the bands in other species/breeds, the same GMM approach can be repeated. In addition, we used data from healthy, free-moving animals. Thus, it might be necessary to adapt the parameters of this analysis for animals during exercise, after drug injection, anesthesia, if genetically modified, or during sleep. Because, the GMM approach is generic, the bands can be easily redefined for other mammalian species or breeds by using the same approach on a representative dataset of animals in these conditions. The ULF band (below 0.003 Hz for humans) has been studied for long term ECG recordings. However, in this work we only consider short segments of no longer than 5 min. As a result, we cannot resolve frequencies below 0.0033 Hz [=1/(60^∗^5)]. For that reason, we defined the VLF band as starting at 0.0033 Hz for humans, dogs, and rabbits, and 0.0056 Hz [=1/(60^∗^3)] for mice. Finally, a better-adapted window size could improve the accuracy of the bands when using the GMM approach, particularly in the case of dogs and rabbits, where this problem has not been studied. However, when the GMM approach was tested with a 3 min or 5 min window on the mouse data, the cutoff frequencies between the bands were in the same range: f_VLF-LF_ = 0.18 and f_LF-HF_ = 1.33 with a 5 min window versus f_VLF-LF_ = 0.15 and f_LF-HF_ = 1.24 with a 3 min window.

## Author Contributions

JB, AR, and YY conceived and designed the research. GK, KM, GB, and OS recorded and formatted the data. JB and AR performed data analysis. JB and YY drafted the manuscript. JB, YY, AR, GB, OS, GK, and KM critically revised the manuscript. All the listed authors qualify for authorship and approved the final version of the manuscript.

## Conflict of Interest Statement

The authors declare that the research was conducted in the absence of any commercial or financial relationships that could be construed as a potential conflict of interest.

## Abbreviations

BM: body mass
BM_m_: typical body mass of a given mammal
BRPM: breaths per minute
ECG: electrocardiogram
GMM: Gaussian mixture model
HF: high frequency band of the power spectral density
HR: heart rate
HR_m,_: typical heart rate of a given mammal
HRV: heart rate variability
LF: low frequency band of the power spectral density
NN: filtered RR interval
NSR: normal sinus rhythm
PSD: power spectral density
RR: beat to beat interval derived from the ECG signal
ULF: ultra-low frequency
VLF: very low frequency band of the power spectral density
