# Supplementary material for: A Universal Scaling Relation for Defining Power Spectral Bands in Mammalian Heart Rate Variability Analysis
Source: Front Physiol. 2018 Aug 2;9:1001. doi: 10.3389/fphys.2018.01001 (PMC6083004; doi:10.3389/fphys.2018.01001)
Supplement: Supplementary file 1 [file Table_1.DOCX]

**A universal scaling relation for defining power spectral bands in mammalian heart rate variability analysis**

Running title: Power spectral bands in mammals

Joachim A. Behar^#,1^, Aviv A. Rosenberg^#,1^, Ori Shemla^1^, Kevin R. Murphy^2^,

Gideon Koren^2^, George E Billman^3^, and Yael Yaniv^*,1^

^1^Biomedical Engineering Faculty, Technion-IIT, Haifa, Israel

^2^Cardiovascular Research Center, Division of Cardiology, Rhode Island Hospital, Warren Alpert Medical School of Brown University, Providence, Rhode Island

^3^Department of Physiology and Cell Biology, The Ohio State University, Columbus, Ohio

^#^JB and AR have contributed equally to this work.

***Corresponding author:**

Yael Yaniv, PhD

Laboratory of Bioenergetic and Bioelectric Systems, Biomedical Engineering Faculty, Technion—IIT, Haifa, Israel

Email: yaely@bm.technion.ac.il

Telephone: 972-4-8294124

Fax: 972-4-8294599

**SUPPLEMENTAL MATERIAL**

**Additional Figures**


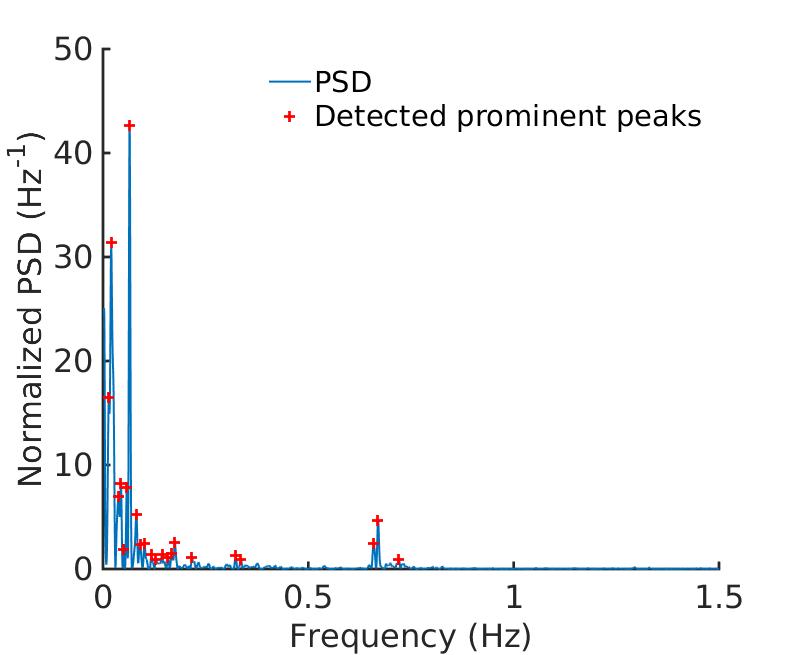


**Figure S1:** Representative example of prominent peaks detected from a 5-min NN interval time series from a rabbit ECG. The red crosses show the prominent detected peaks.


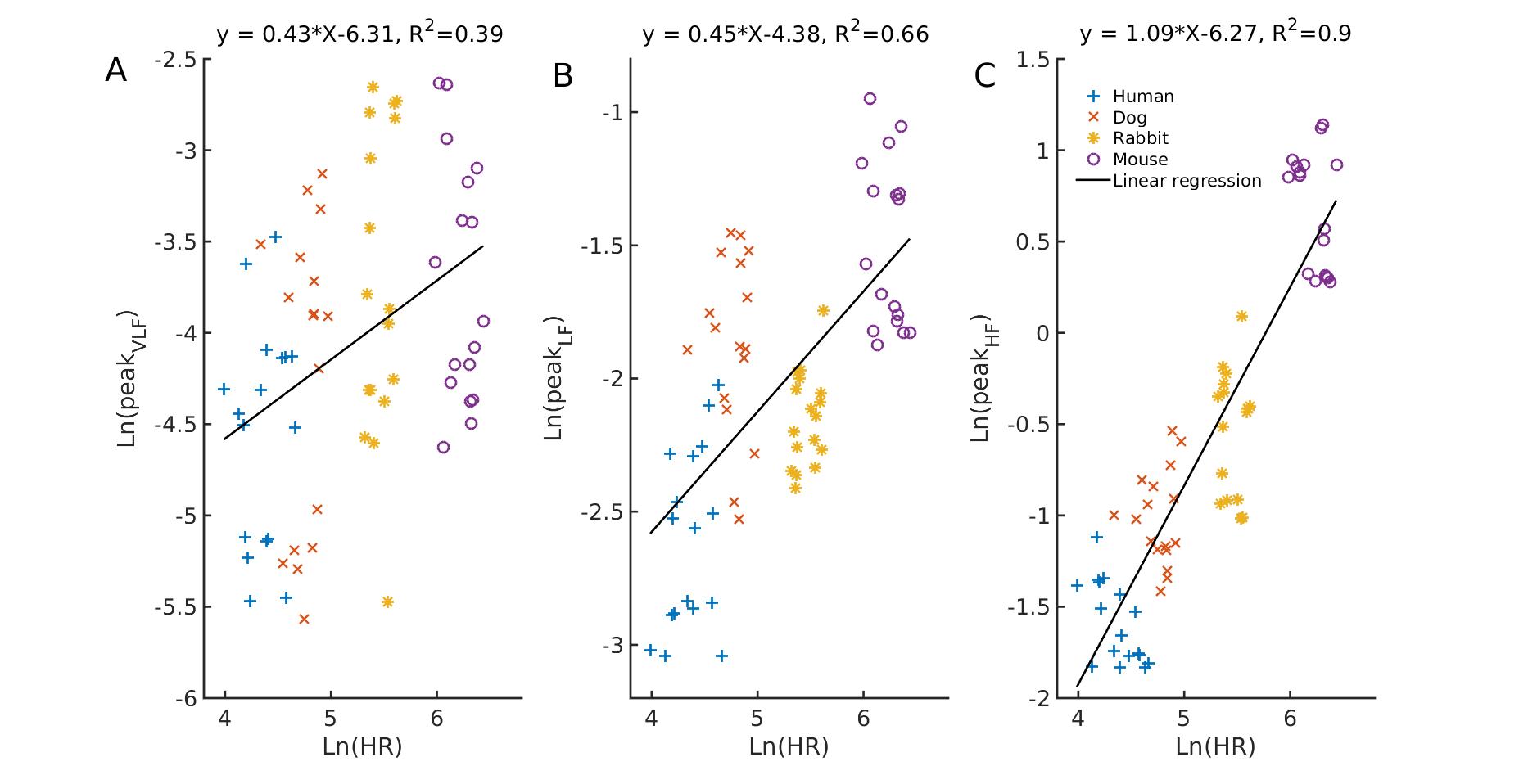


**Figure S2:** Double-logarithmic plot of peak frequency vs. average HR for (A) VLF band, (B) LF band and, (C) HF band.


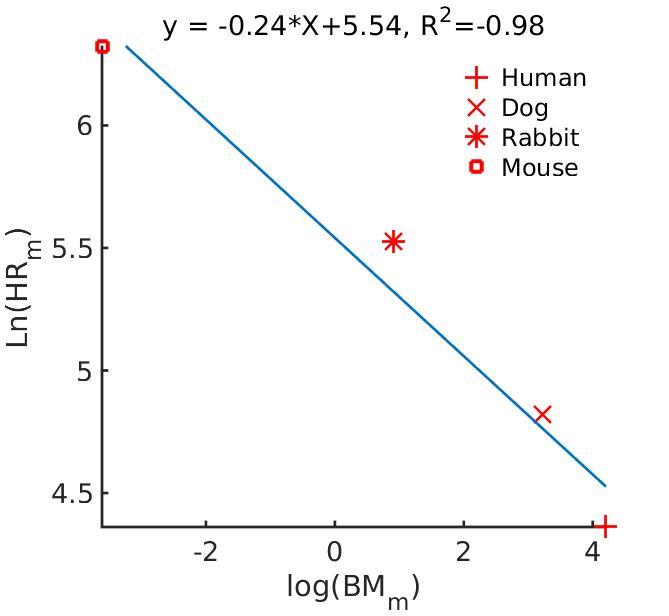


**Figure S3:** Double-logarithmic plot of HR_m_ vs. BM_m_. We retrieve the known allometric law of power -1/4 between HR_m_ and BM_m_.


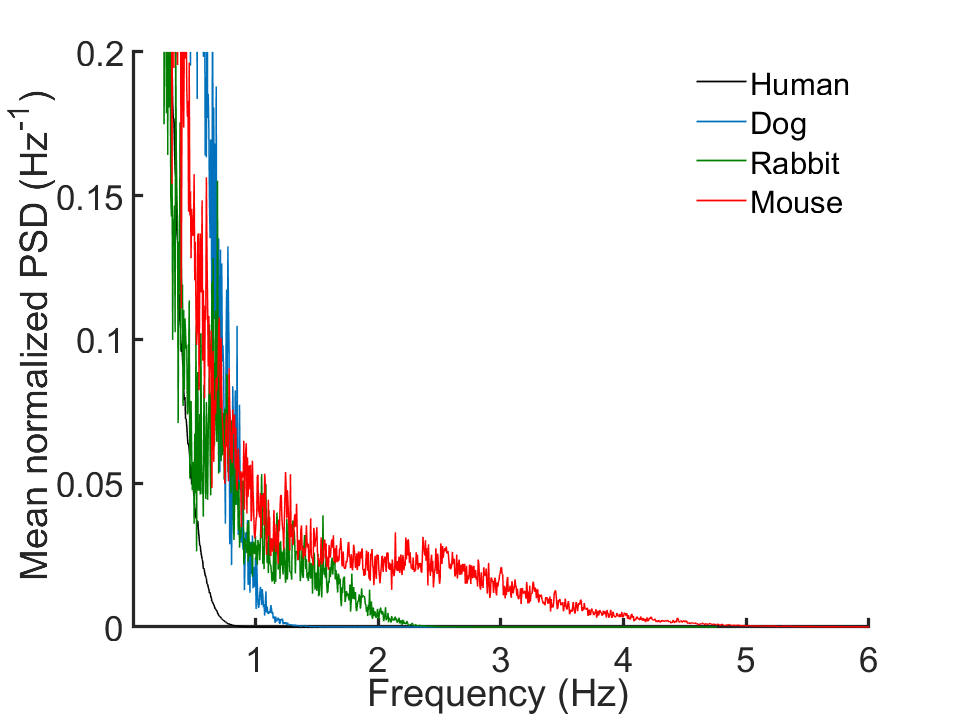


**Figure S4:** Mean normalized power spectral density for each mammalian database. Note the shift of the high frequency content to the right as the mammalian size gets smaller (human, dog, rabbit, mouse.) This is due to the fact that the respiratory sinus arrhythmia activity is higher in smaller mammals.

**Additional Tables**

**Table S1**: Dog database with reference annotations

| File name | Length (hr:min:sec) | Total number of windows |
| --- | --- | --- |
| Dog_01 | 00:04:29 | 1 |
| Dog_02 | 00:06:31 | 1 |
| Dog_03 | 00:06:34 | 1 |
| Dog_04 | 00:04:52 | 1 |
| Dog_05 | 00:05:54 | 1 |
| Dog_06 | 00:05:35 | 1 |
| Dog_07 | 00:04:37 | 1 |
| Dog_08 | 00:05:50 | 1 |
| Dog_09 | 00:06:01 | 1 |
| Dog_10 | 00:05:54 | 1 |
| Dog_11 | 00:05:49 | 1 |
| Dog_12 | 00:04:09 | 1 |
| Dog_13 | 00:04:35 | 1 |
| Dog_14 | 00:06:48 | 1 |
| Dog_15 | 00:05:33 | 1 |
| Dog_16 | 00:04:44 | 1 |
| Dog_17 | 00:06:01 | 1 |
| Total | 01:33:55 | 17 |

**Table S2:** Rabbit database with reference annotations

| File name | Length (hr:min:sec) | Total number of windows |
| --- | --- | --- |
| Rabbit_01_part_1 | 00:15:41 | 3 |
| Rabbit_01_part_2 | 00:04:59 | 1 |
| Rabbit_01_part_3 | 00:04:53 | 1 |
| Rabbit_01_part_4 | 00:07:45 | 1 |
| Rabbit_01_part_5 | 00:12:22 | 2 |
| Rabbit_02_part_1 | 00:05:39 | 1 |
| Rabbit_02_part_2 | 00:08:23 | 1 |
| Rabbit_02_part_3 | 00:09:04 | 1 |
| Rabbit_02_part_4 | 00:26:00 | 5 |
| Rabbit_02_part_5 | 00:13:12 | 2 |
| Rabbit_03_part_1 | 00:05:16 | 1 |
| Rabbit_03_part_2 | 00:07:46 | 1 |
| Rabbit_03_part_3 | 00:06:52 | 1 |
| Rabbit_03_part_4 | 00:05:59 | 1 |
| Rabbit_03_part_5 | 00:14:54 | 2 |
| Rabbit_03_part_6 | 00:13:48 | 2 |
| Rabbit_03_part_7 | 00:08:21 | 1 |
| Rabbit_04_part_1 | 00:18:27 | 3 |
| Rabbit_04_part_2 | 00:15:23 | 3 |
| Rabbit_04_part_3 | 00:06:28 | 1 |
| Total | 03:31:13 | 34 |

**Table S3:** Mouse database with reference annotations

| File name | Length (hr:min:sec) | Total number of windows |
| --- | --- | --- |
| Mouse_19 | 00:19:20 | 6 |
| Mouse_20 | 00:13:53 | 4 |
| Mouse_21 | 00:29:20 | 9 |
| Mouse_22 | 00:33:18 | 11 |
| Mouse_23 | 00:29:38 | 9 |
| Mouse_24 | 00:20:40 | 6 |
| Mouse_25 | 00:22:20 | 7 |
| Mouse_26 | 00:39:38 | 13 |
| Total | 03:28:07 | 65 |
